# Galacto-oligosaccharides fed during gestation increase Rotavirus A specific antibodies in sow colostrum, modulate the microbiome, and reduce infectivity in neonatal piglets in a commercial farm setting

**DOI:** 10.3389/fvets.2023.1118302

**Published:** 2023-02-07

**Authors:** Adam Lee, Lu Liang, Phillippa L. Connerton, Ian F. Connerton, Kenneth H. Mellits

**Affiliations:** Division of Microbiology, Brewing, and Biotechnology, School of Biosciences, Sutton Bonington Campus, University of Nottingham, Loughborough, Leicestershire, United Kingdom

**Keywords:** rotavirus, microbiota, pigs, galacto-oligosaccharides, antibodies, colostrum

## Abstract

**Introduction:**

Rotavirus A is a major cause of acute dehydrating diarrhea in neonatal pigs resulting in significant mortality, morbidity, reduced performance and economic loss. Commercially available prebiotic galacto-oligosaccharides are similar to those of mammalian milk and stimulate the development of the microbiota and immune system in neonates. Little is known about the effects of supplementing sows' diets with galacto-oligosaccharides during gestation. This study aimed to determine if dietary galacto-oligosaccharide supplementation during gestation could improve immunity, reduce rotavirus infection and modulate the microbiota in sows and neonates in a commercial farm setting with confirmed natural endemic rotavirus challenge.

**Methods:**

In a randomized controlled trial, control sows received lactation diet with no galacto-oligosaccharide supplementation and test sows received lactation diet with 30 g/day galacto-oligosaccharide top-dressed into feed daily, seven days before farrowing. Colostrum was collected from sows 24 hours *post-partum* and tested for rotavirus specific antibodies. Fecal samples were collected from sows and piglets three days *post-partum*, tested for rotavirus A by qPCR and the microbiome composition assessed by 16s rRNA gene sequencing.

**Results:**

Supplementation with galacto-oligosaccharides during gestation significantly increased rotavirus-specific IgG and IgA in sow colostrum and reduced the number of rotavirus positive piglet fecal samples. Abundance of potential pathogens *Treponema* and *Clostridiales* were higher in fecal samples from non-galacto-oligosaccharide fed sows, their piglets and rotavirus positive samples.

**Discussion:**

This study demonstrates that galacto-oligosaccharide supplementation during gestation significantly increases rotavirus specific IgG and IgA in sow colostrum thereby reducing neonatal rotavirus infection and suppresses potential pathogenic bacteria in nursing sows and neonatal piglets.

## Introduction

Rotaviruses are classified into at least ten serogroups (1, 2) with A, B, and C affecting humans (3), whilst groups A to H have been found in pigs (2). The most common groups are A, B and C, with Rotavirus A (RVA) representing the most prevalent group causing acute dehydrating diarrhea in public and veterinary health settings (2). RVA fecal-oral infection results in destruction of small intestinal enterocytes, the development of malabsorptive diarrhea (4) and promotes gut dysbiosis through alteration of the microbiota (5).

The effects on pigs are significant mortality and morbidity in neonates, reduced performance in surviving growers and significant economic loss (1, 2, 6). RV is endemic in UK pig farms. A range of RVA genotypes has been identified in UK pigs: six G types (VP7); G2, G3, G4, G5, G9, and G11 and six P types (VP4); P6, P7, P8, P13, P23, and P32 (7). Furthermore, the common human genotype P8 can infect pigs highlighting the need for surveillance of porcine rotavirus genotypes to safeguard human and porcine health (7).

Previous livestock vaccination strategies have focussed on the induction of active (immune cell mediated) and passive (antibody mediated) immunity by oral administration of attenuated RV vaccines (8). However, these have lacked efficacy, in contrast to engineered virus-like particles (VLP) designed as vaccines to boost antibodies in bovine and porcine mammary secretions which have shown promise when administered with attenuated vaccines (9). The wide variety of RV genotypes in pigs complicates effective vaccine production. This is further complicated by attenuated replicating porcine RVA vaccines which may contribute to the diversity of porcine RVs, through re-assortment of vaccine strains with wild type strains and the emergence of novel genetic variants that can evade herd immunity (2, 7). Whilst vaccination remains popular in the farming community, a more pragmatic view may be to focus on cleaning and disinfection with efficacious detergents that not only limit the spread and infectivity of RV but also other microbial pathogens (10, 11). Nevertheless, endemic porcine RV infection still needs alternative strategies to boost lactogenic immunity in sows, thus providing RV antibodies to the neonate with colostrum and milk (2).

Galacto-oligosaccharides (GOS) are a major constituent of mammalian milk (12, 13) primarily stimulating the development of the microbiota in neonates and conferring a variety of health benefits including innate and adaptive immune development (14, 15). Milk oligosaccharides are typically composed of three to ten monosaccharide units, including glucose (Glc), galactose (Gal) and N-acetyl-glucosamine (GlcNAc) as well as fucose and sialic acids. The core moiety present at the reducing end of milk oligosaccharides is either lactose (Gal(β1–4)Glc) or N-acetyl-lactosamine (Gal(β1–4)GlcNAc) (16). Most animal milk oligosaccharides are sialylated, containing N-acetylneuraminic acid (Neu5Ac) and/or N-glycolylneuraminic acid (Neu5Gc) (17). Compared with other domestic animals, porcine milk contains the highest percentage of neutral oligosaccharides (20%), the most abundant variety of mono-sialylated and di-sialylated large oligosaccharides and are the closest to human milk oligosaccharide composition (13). In addition, porcine milk oligosaccharides (PMOs) decrease in abundance by ~43% during the first week of lactation with the relative concentration of acidic PMOs decreasing and neutral PMOs increasing (18), indicating a change in functionality during lactation.

In pigs there is evidence that GOS is readily fermented in the gastrointestinal tract (GIT) increasing short-chain fatty acid (SCFA) concentrations and increasing beneficial probiotic bacteria numbers (19, 20). Furthermore, GOS may reduce adhesion of pathogens to cells, (21) inhibit pathogen colonization (21), improve gut architecture (20) and reduce expression of pro-inflammatory cytokines (22). Specific effects of GOS on RVs have been demonstrated. For example, GOS/fructo-oligosaccharide mixtures reduce RV induced diarrhea and modulate dysbiosis in suckling rats (5, 23). Human milk oligosaccharides (HMOs) inhibit RV infectivity *in vitro* (24, 25), in acutely infected piglets (24) and reduce the duration of RV-induced diarrhea in piglets whilst modulating the colonic microbiota *in vivo*. (26). Also, RV specific antibodies from Human breast milk neutralize RV infectivity *in vitro* (27). However, most studies have focussed on feeding neonatal to pre-weaning piglets GOS, whilst few have considered supplementing the diets of gestational sows to determine effects on the neonate. It has been reported that the combination of GOS and casein glycomacropeptides (CGMP) fed to gestational sows modulated the neonatal microbiota colonization, promoted gut development and growth performance of piglets, thus demonstrating that manipulation of the maternal gestational immune/microbiome axis has positive effects on offspring, but without RVA challenge (28). The aims of this study were to determine if GOS supplementation in gestational sows conferred immunity, reduced infectivity and modulated the microbiome in neonatal piglets in a commercial pig farm where RV challenge is naturally endemic and as confirmed by previous veterinary reports.

## Materials and methods

### Experimental design

#### Animals

A randomized controlled trial was performed on a commercial farrow-to-finish pig farm in Yorkshire UK, between October and December 2018. The trial was approved by the farm veterinary consultant and by the University of Nottingham ethics committee on 12-9-18, approval reference number 190. Landrace x Large white sows crossed with a Piétran boar were paired with respect to parity. Gestating sows of similar weight were moved to 3.0 × 1.8 m farrowing pens with a 0.8 × 2.2 m farrowing crate, seven days before farrow. Pens had a slatted floor and were heated with industry standard heat lamps. Temperature was kept at range 18–20°C for sows and 23–24°C for piglets with light periods from 8:00 am to 17:00 pm. Relative humidity was 50 to 70% for farrowing units and 24 to 30% for weaning units. Metal chain toys with plastic balls were provided as environmental enrichment. Sows received a wheat-based lactation diet (Gold Lactator, Noble Foods, Stokesley, UK) containing 18.4% protein, 5.6% ash, 4.6% oil, 4.1% fiber, 1.13% lysine, 0.9% calcium, 0.34% methionine and 0.49% phosphorous. New-born pigs received a 1 ml intramuscular iron injection (Gleptosil, Alstoe Ltd, York, UK) 24 h after birth. Sows and gilts were vaccinated with a combined Rotavirus OSU 6 strain and *E. coli* strains 0101:K99 vaccine two weeks prior to farrowing as per manufacturer's instructions and as according to standard farm practice (Rokovac Neo, Bioveta, Czech Republic). Piglets and sows did not receive any creep feed supplementation or prophylactic antibiotic treatment during the trials. Sows were individually housed and randomized in a homogenous pattern to either basal control diet or supplementation with GOS powder (DP2+ GOS, Nutrabiotic, Saputo Dairy UK, Weybridge UK). Sows received the lactation diet with no GOS supplementation (non-GOS sows) or received the lactation diet with 30 g/day GOS top-dressed into feed daily, seven days before farrowing (GOS sows). Piglets born to non-GOS sows were referred to as non-GOS piglets and those born to GOS sows were referred to as GOS piglets. Trial size was determined using a power calculation accessed at: https://clincalc.com/stats/samplesize.aspx on 02-08-18, where α = 0.05, β = 0.2, and power = 0.8, giving thirty-six replicates per control and treatment groups with a total of seventy-two pens, with one sow per pen. Trials were repeated six times, from week one to week six, in order to obtain the desired number of replicates. Models were fixed effect, whereby sows from the production cohort were randomly allocated to farrowing pens pre-assigned for non-GOS or GOS feed (independent variables). All animals were kept in identical environmental conditions, housed in identical pens and in the same building. Pens were cleaned and disinfected prior to trial replicates from the end of week one to week six onwards throughout the entire standard farm production methods. Once born, neonatal piglets were cross fostered within treatment groups, as per commercial farm standard practice, to equilibrate litter size and for welfare reasons. All trial personnel, including investigators were blinded to treatment allocation. All animals were monitored daily by trained farm personnel for any signs of scour, disease, lameness and/or distress. No animals were euthanized, or invasive samples taken during studies.

### Sample collection

Trained farm personnel collected samples for biosecurity reasons. Colostrum from sows was collected within 24 hours post parturition by massaging the two teats closest to the head of sows and immediately frozen at −20°C, in a freezer, in 30 ml sterile plastic universal tubes (Thermo Scientific, Loughborough, UK). Approximately 2–3 g of freshly voided fecal samples were collected from sows and piglets per pen, in sterile nuclease free 2 ml micro tubes (Sarstedt, Leicester, UK) three days post partition and immediately frozen at−20°C. Fecal samples from piglets were pooled from each pen, whilst those of sows were kept separately. Frozen samples were delivered by refrigerated courier service to the University of Nottingham for storage at −80°C and further laboratory analyses.

### ELISA for RVA IgG and IgA in colostrum

Samples were defrosted and 1 ml aliquots centrifuged at 13,000 g for 15 min to separate the fat from the colostrum. Aqueous phase colostrum was pipetted from underneath the fat layer and into sterile 2 ml micro tubes for subsequent analysis. The Ingezim rotavirus porcine ELISA kit (Immunologia Y Genetica Aplicada S.A. Madrid, Spain) was used to determine specific anti-RVA IgG and anti-RVA IgA activity in the colostrum samples according to manufacturer's instructions. For the detection of anti-RVA IgA antibodies, ELISA was performed as with IgG, but the secondary antibody was substituted with peroxidise-labeled goat anti-porcine IgA (Thermo Fisher Scientific, Bonn, Germany) at a dilution of 1/10,000 as according to Kreuzer et al. (29). The positive control serum supplied with the kit, was assayed on each occasion and the mean value from these measurements used to obtain a normalized absorbance ratio to reduce assay-to-assay variation (30). Total non-specific IgG and IgA in colostrum were assayed using IgG and IgA Pig ELISA Kits obtained from (Abcam plc, Cambridge, UK).

### DNA and RNA extraction

Bacterial DNA was extracted from 200 mg sow and piglet fecal samples using the QIAamp PowerFecal QIAcube HT Kit and QIAcube HT robot according to manufacturer's instructions (Qiagen, Hilden, Germany). Viral RNA was extracted from sow and piglet feces by mixing 100 mg with 900 μl isotonic 0.9% NaCl (Merck, Gillingham, UK), prepared in diethyl pyrocarbonate (DEPC) treated nuclease free water (Fisher Scientific UK Ltd, Loughborough UK), vortexed and centrifuged at 16,000 g for 5 min. All glassware was treated with 0.1% v/v DEPC (Merck, Gillingham, UK), to remove RNase enzymes and autoclaved at 121°C at 15 psi to eliminate residual DEPC. 200 μl of the clear supernatant was used for viral nucleic acid extraction using the QIAamp 96 Virus QIAcube HT Kit and QIAcube HT robot according to manufacturer's instructions (Qiagen, Hilden, Germany). DNA was digested in samples by including an optional DNase digestion step in the QIAcube HT protocol using the Qiagen RNase Free DNase Set (Qiagen, Hilden, Germany) to prevent the possibility of interference with RNA assays in downstream applications. Bespoke software for loading onto the QIAcube HT robot was provided by Qiagen for this step. During viral RNA extraction 4 μl per sample of a Techne qPCR Rotavirus A kit internal extraction control RNA was spiked into the lysis buffer as a positive control for the extraction process (Cole-Parmer, Stone, Staffordshire UK).

### Detection of RVA RNA in RNA samples

The Techne qPCR Rotavirus A kit was used to detect the presence of RVA in samples with an amplification protocol using OneStep 2x Reverse Transcription-qPCR MasterMix according to manufacturer's instructions (Cole-Parmer, Stone, Staffordshire UK). RVA specific primer probe mix was used to detect the presence of RVA non-structural protein 5 (NSP5) genomes. Standard curves were prepared with RVA positive control template with copy numbers from 2 × 10^5^ per μl to 2 per ul. Real-time quantitative PCR data were collected using the Roche LightCycler 480 (Hoffman La Roche, Basel, Switzerland). The amplification protocol was reverse transcription for 10 min at 42°C, enzyme activation for 2 min at 95°C, then 50 cycles of denaturation for 10 s at 95°C and fluorogenic data collection for 60 s at 60°C followed by one cycle of cooling. The detection format was dual color hydrolysis/Universal Probe Library (UPL), with dynamic integration time mode and a filter combination of duplexing TaqMan probes, FAM and VIC. Amplification curves were initially analyzed using the LightCycler 480 Software release 1.5.0.39. as obtained from https://pim-eservices.roche.com/eLD/web/gb/en/products/3.8.1.4.4.8 accessed 20-02-20.

### PCR amplification of 16S rRNA gene sequences

Using the extracted DNA as a template, the V4 region of the bacterial 16S rRNA genes were PCR amplified using primers 515f (5' GTGCCAGCMGCCGCGGTAA 3') and 806r (5' GGACTACHVGGGTWTCTAAT 3') (31). The full preparation and sequencing of 16S rRNA gene sequencing libraries were conducted according to the MiSeq Wet Lab SOP accessed at https://github.com/SchlossLab/MiSeq_WetLab_SOP/blob/master/MiSeq_WetLab_SOP on the 19-02-20. Amplicons were sequenced on the Illumina MiSeq platform (Illumina, San Diego, CA, USA) using 2 × 250 bp cycles (32). Sequence data were deposited in the NCBI database within Bioproject PRJNA884280.

### Microbiota diversity analysis

The 16S rRNA sequence analyses were performed using Mothur v. 1.43, (33) open source software and accessed at: (https://github.com/mothur/mothur/releases accessed 12-03-20). Analysis was performed according to the MiSeq SOP accessed at: (https://mothur.org/wiki/miseq_sop/ accessed 12-03-20). The 16S rRNA gene sequences were aligned against a reference alignment based on the SILVA rRNA database for use in Mothur available at: (https://mothur.org/wiki/silva_reference_files accessed 12-03-20) (34) and clustered into OTUs using the “opticlust” clustering algorithm (35). The consensus taxonomy of the OTUs was generated using the “classify.otu” command in Mothur with reference data from the Ribosomal Database Project (version 14) (36, 37) adapted for use in Mothur available at: (https://mothur.org/wiki/rdp_reference_files accessed 12-03-20).

### Statistical analyses

Analyses were performed in R version 4.1.1 using R Studio (2021.09.0) (38) unless otherwise stated. Shapiro Wilk tests (39) were used to determine normality for the results of ELISA, log_10_ copy numbers for RVA positive fecal samples and microbiota α-diversity metrics. For ELISA and log_10_ copy numbers of RVA positive samples, significant differences between groups were tested using Mann–Whitney *U*-tests. Significant differences in the number of RVA infected piglet fecal samples were tested using the Binomial test. Coverage and α-diversity expressed as Inverse Simpson diversity (40), Chao (41) Richness, Shannon (42) Index, and ACE Estimator (43), were calculated using the “summary.single” command in Mothur (33). Significant differences were tested for using Kruskal–Wallis rank sum tests. Estimates of β-diversity were calculated in Mothur as Yue and Clayton (44) Dissimilarity (θ_YC_), Bray and Curtis (45) Dissimilarity and Jaccard (46) Similarity. Analysis of molecular variance executed in Mothur (AMOVA) was used to test for differences in β-diversity between samples (47, 48). Linear discriminant analysis effect size (LEfSe) was used to examine differential OTU abundances at genus level in Mothur (49). Where appropriate, multiple comparisons (AMOVA, Kruskal–Wallis rank sum tests) were adjusted for false discovery rates (FDR) by the Benjamini and Hochberg procedure (50) (*P* = 0.05, FDR = 25%).

## Results

### RVA specific and total antibody titres in sow colostrum

**Figure 1:**
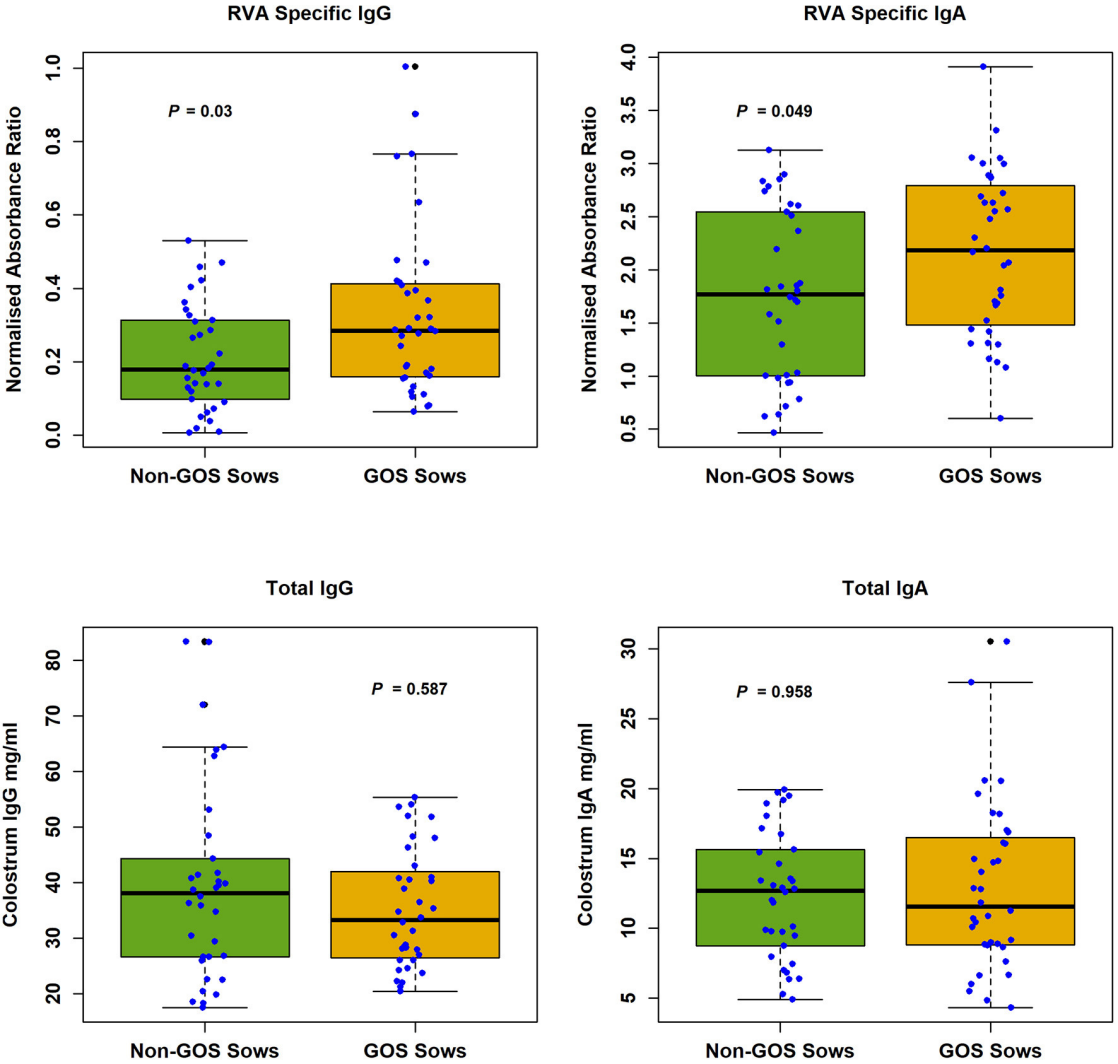
RVA specific IgG and IgA and total IgG and IgA in colostrum from non-GOS and GOS fed sows.

RVA specific and total antibody levels in sow colostrum are shown in Figure 1. Median RVA specific antibody levels in sow colostrum were, IgG non-GOS sows 0.179, IgG GOS sows 0.285, IgA non-GOS sows 1.771, IgA GOS sows 2.182 (normalized absorbance ratios). Median total antibody levels in sow colostrum were, IgG non-GOS sows 38.06, IgG GOS sows 33.25, IgA non-GOS sows 12.83, IgA GOS sows 11.55 (mg/ml colostrum). Shapiro-Wilk normality tests indicated colostrum concentrations of RVA specific and non-specific antibodies were not normally distributed (*P* < 0.05 in each case). Colostrum RVA specific IgG and IgA concentrations expressed as ELISA normalized absorbance ratio were significantly higher in GOS fed sows compared with non-GOS sows (*P* = 0.03 and *P* = 0.049 respectively, Mann–Whitney *U-*tests). However, total IgG and IgA colostrum contents were not significantly different between GOS fed sows compared with non-GOS sows (*P* = 0.587 and *P* = 0.886 respectively, Mann–Whitney *U-*tests) (Figure 1).

### qPCR identification of RVA infected fecal samples

Internal extraction control RNA spiked into lysis buffer during viral RNA extraction was positive for all samples indicating successful RNA extraction and qPCR amplification using the LightCycler 480 VIC channel. Log_10_ copy numbers per g of fecal material for RVA positive samples from non-GOS piglets and GOS piglets were non-normally distributed (*P* = 5.7 × 10^−4^ and *P* = 0.024, respectively using Shapiro–Wilk tests). Median log_10_ copy numbers per g of fecal material were 16.25 for non-GOS piglets and 17.12 for GOS piglets. There was no significant difference in the RVA log_10_ copy number between non-GOS piglets or GOS piglets (*P* = 0.7007, Mann–Whitney *U-*tests). Out of thirty-four non-GOS piglet fecal samples, twelve (35%) tested negative and twenty-two (65%) positive for RVA. Out of thirty-six GOS piglet fecal samples, twenty (55%) tested negative and sixteen (45%) positive for RVA. There was a significant difference in the number of piglet fecal samples testing RVA positive between groups, *P* = 0.0085, Binomial test. Out of seventy-one sow fecal samples analyzed seven proved RVA positive, four non-GOS sow fecal samples (8.15–14.23 log_10_ copy number per g) and three GOS sow fecal samples (7.72–11.75 log_10_ copy number per g).

### Fecal microbiota diversity and composition

**Figure 2:**
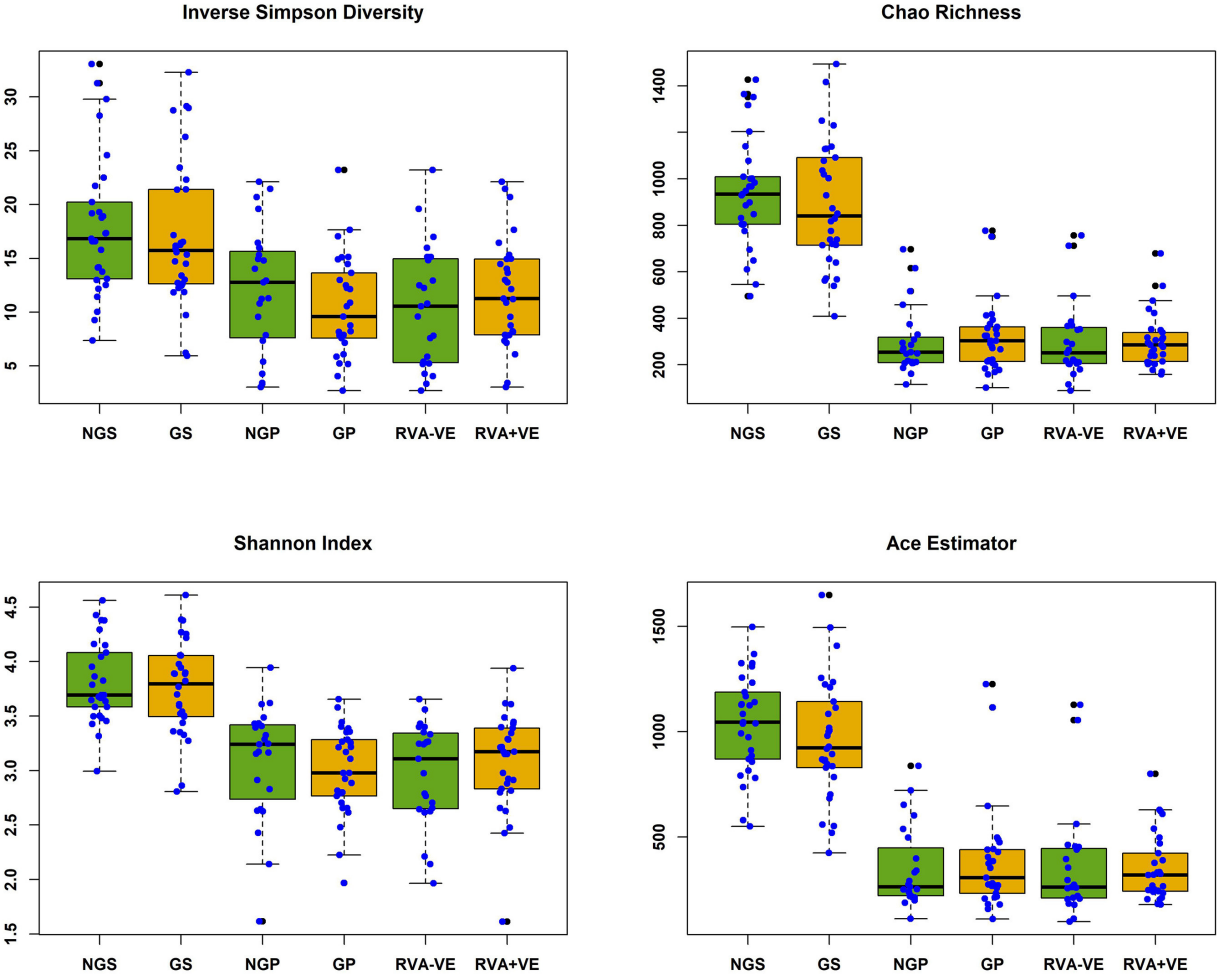
Alpha diversity of fecal samples collected from sows and piglets during suckling. NGS, non-GOS fed sow; GS, GOS fed sow; NGP, non-GOS piglet; GP, GOS piglet; RVA-VE, RVA negative piglet; RVA+VE, RVA positive piglet. Significant difference between NGS and NGP, GS, and GP in all cases *P* < 0.005.

**Figure 3:**
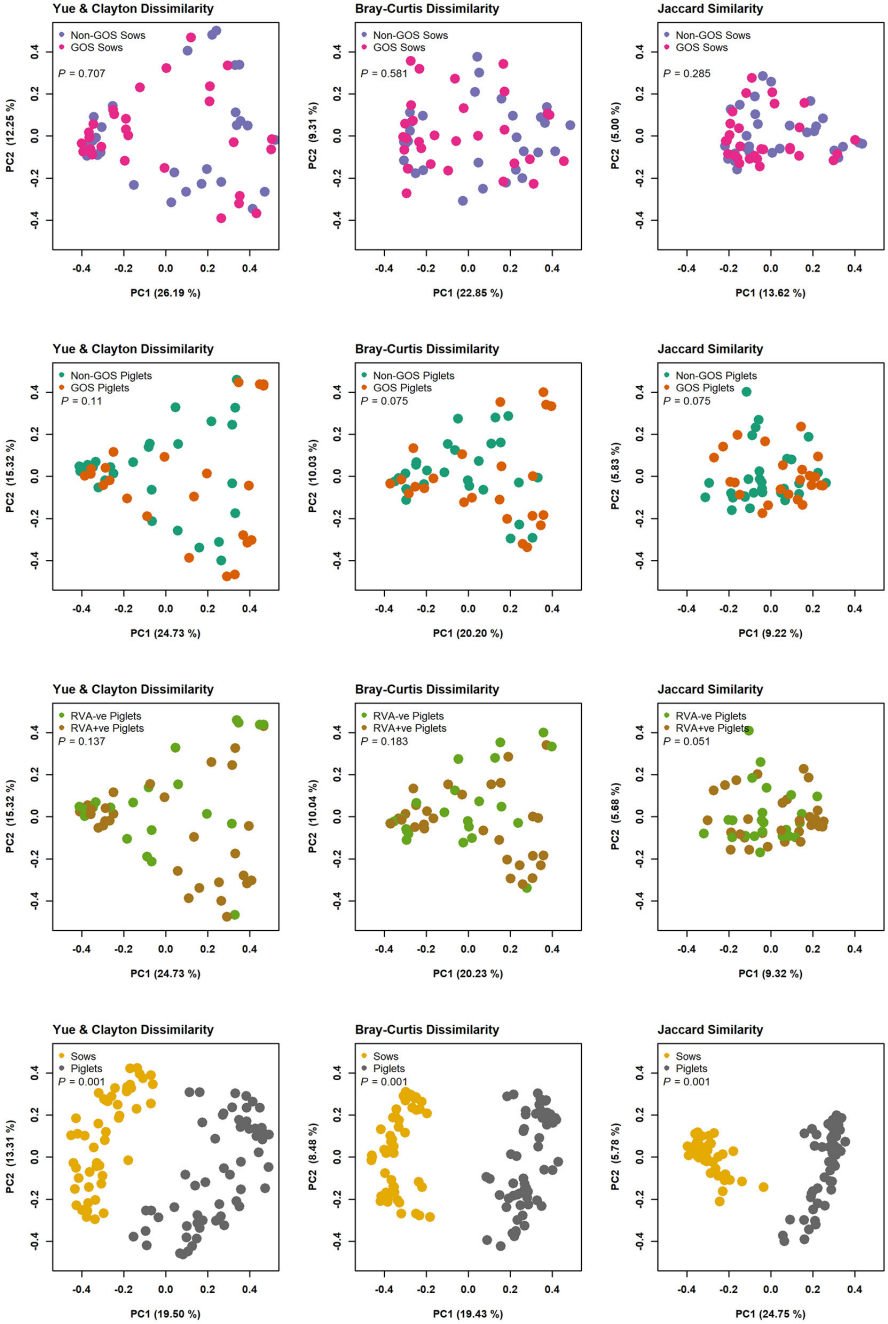
β-diversity measures for fecal samples from non-GOS sows vs. GOS fed sows, non-GOS piglets vs. GOS piglets, RVA negative vs. RVA positive piglets and sows vs. piglets.

In total 3,333,385 high quality 16S rRNA, V4 sequences were obtained from 141 sow and piglet fecal samples. Of these, 2,189,090 were recovered from seventy-one sow fecal samples and 1,144,295 from seventy piglet fecal samples. By treatment groups, 1,021,516 sequences were recovered from thirty-five non-GOS fed sows, 1,167,574 from 36 GOS fed sows, 449,463 from thirty-four piglets born to non-GOS fed sows and 694,832 from thirty-six piglets born to GOS fed sows. Sequences were subsampled to 11,210 per sample with a Good's coverage (51) of 97.8 to 99.9%. Metrics for α-diversity were not normally distributed (Shapiro–Wilk tests). There were no significant differences in α-diversity metrics between non-GOS fed sows and GOS fed sows, or piglets born to non-GOS fed sows and piglets born to GOS fed sows, *P* > 0.05 in each case (Kruskal–Wallis rank sum tests). α-diversity for all four metrics were significantly higher in non-GOS sows as opposed to non-GOS piglets and GOS sows as opposed to GOS piglets *P* < 0.005 in each case (Figure 2). Calculated β-diversity θ_YC_, Bray and Curtis (45) and Jaccard (46) distances between non-GOS fed sows and GOS fed sows were not significantly different, as determined by AMOVA (48), *P* = 0.707, *P* = 0.581, and *P* = 0.285, respectively. θ_YC_, Bray-Curtis and Jaccard distances were not significantly different between non-GOS piglets and GOS piglets, *P* =0.11, *P* = 0.102, and *P* = 0.075. There was a highly significant difference between sows and piglets for all three β-diversity metrics, *P* < 0.001 in each case (Figure 3).

**Figure 4:**
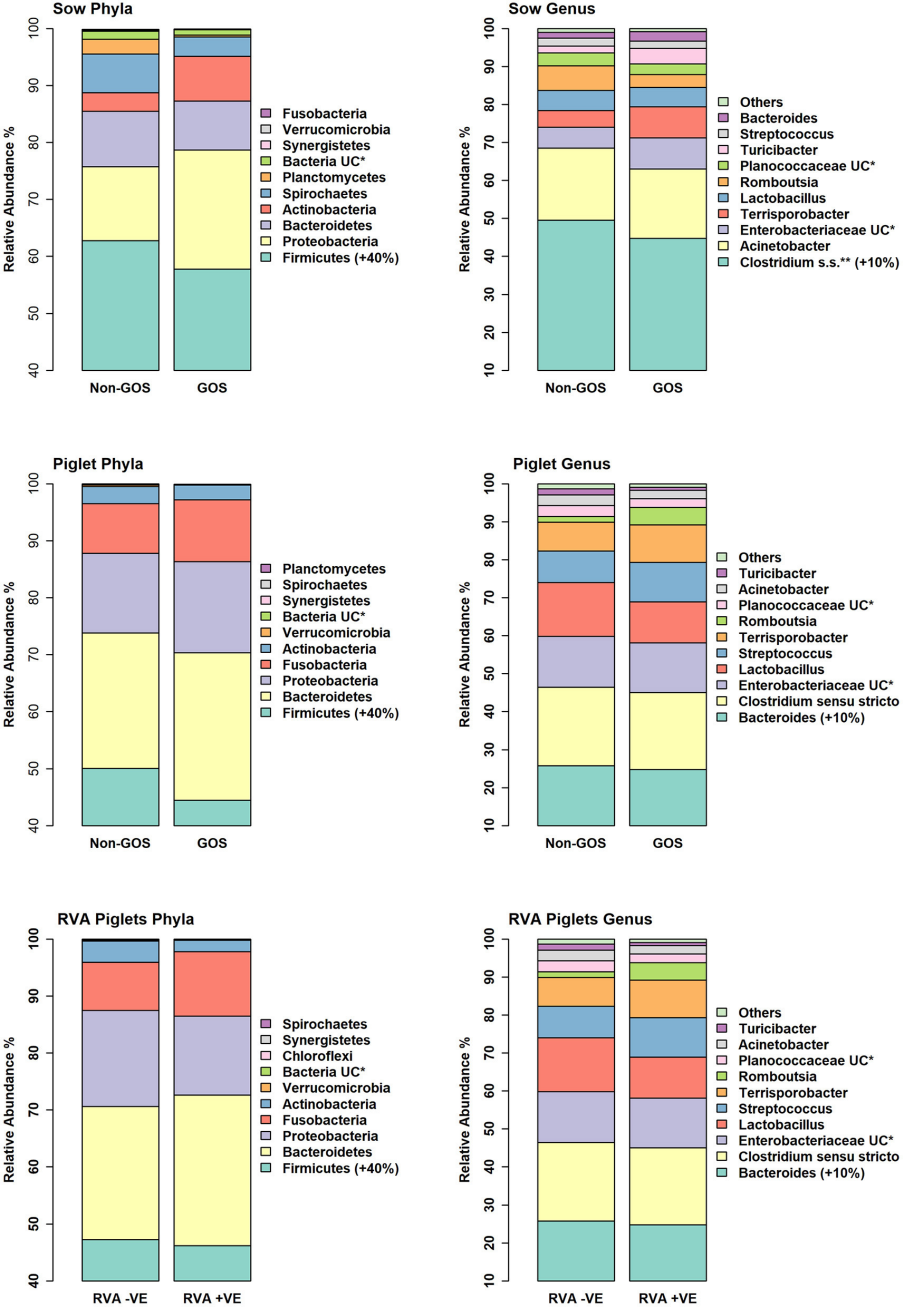
Relative abundance of bacterial taxa at phylum and genus level for fecal samples from non-GOS sows, GOS sows, non-GOS piglets, GOS piglets, RVA negative piglets, and RVA positive piglets.

**Figure 5:**
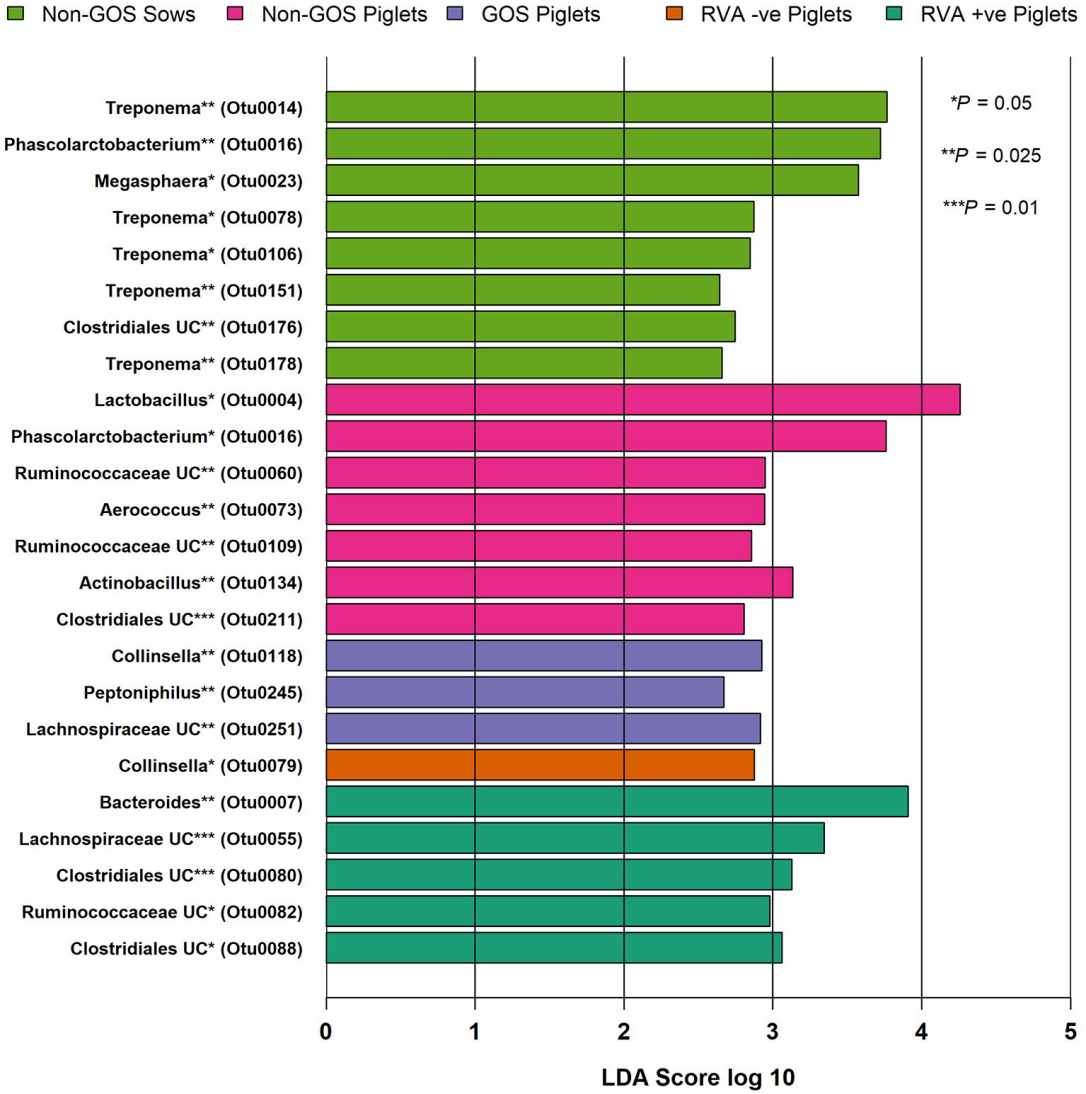
Significant differences in differential abundance of taxa at genus level for fecal samples from non-GOS sows, non-GOS piglets, GOS piglets, RVA negative piglets, and RVA positive piglets (LEfSe). No attributable differential abundance features for GOS sows, therefore not shown. UC = unclassified.

Figure 4 shows relative abundance of bacterial taxa at phylum and genus level for fecal samples from non-GOS sows, GOS sows, non-GOS piglets and GOS piglets. For sow fecal samples, sequences were clustered into 5629 OTUs and classified into 19 unique phyla, 43 classes, 80 orders, 171 families and 397 genera. In total, the top ten taxa allocated to OTUs at phylum level were *Firmicutes* (60.09%), *Proteobacteria* (17.23%), *Bacteroidetes* (9.10%), *Actinobacteria* (5.71%), *Spirochaetes* (4.98%), *Planctomycetes* (1.37%), Bacteria unclassified (1.18%), *Synergistetes* (0.11%), *Verrucomicrobia* (0.06%) and *Fusobacteria* (0.03%). The top ten taxa allocated to OTUs at genus level were, *Clostridium sensu stricto* (18.63%), *Acinetobacter* (6.89%), *Enterobacteriaceae* unclassified (6.43%), *Terrisporobacter* (5.19%), *Lactobacillus* (4.85%), *Romboutsia* (3.07%), *Planococcaceae* unclassified (3.02%), *Turicibacter* (2.02%), *Streptococcus* (1.99%) and *Bacteroides* (0.95%). For piglet fecal samples, sequences were clustered into 2273 OTUs and classified into 19 unique phyla, 40 classes, 73 orders, 154 families and 349 genera. The top ten taxa allocated to OTUs at phylum level were *Firmicutes* (46.66%), *Bacteroidetes* (25.03%), *Proteobacteria* (15.21%), *Fusobacteria* (10.01%), *Actinobacteria* (2.76%), *Verrucomicrobia* (0.14%), Bacteria unclassified (0.09%), *Synergistetes* (0.04%), *Spirochaetes* (0.02%) and *Planctomycetes* (0.002%). The top ten taxa allocated to OTUs at genus level were, *Bacteroides* (20.47%), *Clostridium Senso Stricto* (13.17%), *Enterobacteriaceae* unclassified (12.37%), *Lactobacillus* (8.85%), *Streptococcus* (2.59%), *Terrisporobacter* (0.29%), *Romboutsia* (0.21%), *Planococcaceae* unclassified (12%), *Acinetobacter* (0.11%) and *Turicibacter* (0.05%). LEfSe identified significant differences in the abundance of differential OTUs annotated to taxa at genus level between treatment groups (Figure 5). In total non-GOS sows had eight OTUs occurring at significantly higher relative abundance compared with GOS sows, five of these being *Treponema* and one each to *Phascolarctobacterium, Megasphaera*, and *Clostridiales* unclassified. Non-GOS piglets had seven OTUs occurring at significantly higher relative abundance compared with GOS piglets, two of these being *Ruminococcaceae* unclassified and one each to *Lactobacillus, Phascolarctobacterium, Aerococcus, Actinobacillus*, and *Clostridiales* unclassified. GOS piglets had three OTUs occurring at a differentially higher abundance than non-GOS piglets, these being *Peptoniphilus, Lachnospiriaceae* unclassified, and *Collinsella*.

### Fecal microbiota diversity and composition in non-infected and RVA infected piglets

In separate analyses by Mothur, 1,144,334 high quality 16S rRNA, V4 sequences were obtained from seventy piglet fecal samples. Of these, 531,797 were recovered from thirty-two RVA negative samples and 612,537 from thirty-eight RVA positive samples. Sequences were subsampled to 8078 per sample with a Good's coverage of 97.8 to 99.9%. Metrics for α-diversity were not normally distributed (Shapiro–Wilk tests). There were no significant differences in α diversity (Kruskal–Wallis Rank sum tests) or β-diversity (AMOVA) (48). Sequences were clustered into 2188 OTUs and classified into 19 unique phyla, 40 classes, 74 orders, 157 families and 348 genera. Figure 4 shows relative abundance of bacterial taxa at phylum and genus level for RVA negative and RVA positive fecal samples. In total, relative abundance of the top ten OTUs annotated to taxa at phylum level were *Firmicutes* (46.68%), *Bacteroidetes* (25.03%), *Proteobacteria* (15.21%), *Fusobacteria* (10.01%), *Actinobacteria* (2.76%), *Verrucomicrobia* (0.14%), Bacteria unclassified (0.07%), *Chloroflexi* (0.04%), *Synergistetes* (0.03%), and *Spirochaetes* (0.02%). The top ten OTUs annotated to taxa at genus level were Bacteroides (20.47%), *Clostridium sensu stricto* (13.17%), *Enterobacteriacea* unclassified (12.37%), *Fusobacterium* (9.42%), *Lactobacillus* (8.85%), *Prevotella* (3.14%), *Streptococcus* (2.59%), *Peptostreptococcus* (2.43%), *Enterococcus* (1.20%), and *Phascolarctobacterium* (1.11%). LEfSe identified significant differences in the abundance of differential OTUs annotated to taxa at genus level between RVA negative piglets and RVA positive piglets (Figure 5). RVA negative piglets expressed an increased differential abundance of *Collinsella* in contrast with RVA positive piglets. RVA positive piglets expressed a significant differential abundance in five OTUs, two being ascribed to *Clostridiales* unclassified and three others being *Bacteroides, Lachnospiraceae* unclassified, and *Ruminococcacae* unclassified.

## Discussion

The objectives of this study were to determine if GOS supplementation in gestational sows conferred immunity, reduced infectivity and modulated the microbiome in neonatal piglets in a commercial pig farm where RVA challenge was endemic. Whilst PMOs are expressed naturally in sow colostrum (13), supplementation with GOS top-fed at 30 g per day was associated with significantly increased RVA specific IgG and IgA in sow colostrum (*P* = 0.03 and *P* = 0.049 respectively), but not the expression of total IgG and IgA (Figure 1). The maternal gut microbiome breast axis and the importance of entero-mammary pathways in programming the mammary gland to face the nutritional, microbiological, immunological, and neuroendocrine requirements of the growing infant have been well described in humans (52). However, humans possess a hemochorial placenta whereas pigs have an epitheliochorial placenta (53), one which, in contrast, is a relatively impenetrable barrier to maternal immunoglobulins during gestation, particularly IgG. Thus, piglets are born “agammaglobulinemic” and survival depends on early acquisition of maternal immunity through colostrum (54) before gut closure within 24 to 48 h *post-partum* and reduced intestinal enterocyte ability to sequester immunoglobulins from protein rich colostrum (55). Moreover, colostrum intake is the main determinant of piglet survival through energy provision and immune protection with long-term effects on growth and immunity (56). Few animal studies have investigated how pre- and/or probiotics fed to epitheliochorial pregnant mammals interact with the immune composition of mammary secretions. In dogs, pregnant bitches fed a mixture of fructo-oligosaccharides, mannan-oligosaccharides, *E. faecium* and *L. acidophilus* expressed significantly more IgG, IgM and IgA in colostrum (57). Possible mechanisms are the modulation of immunoglobulin secretion by the maternal microbiome. In murine models, gut microbiome induced maternal IgG is transferred to the neonatal intestine through milk *via* neonatal Fc receptors and directly inhibits pathogen colonization (58). For IgA, the gut microbiota induces Peyer's-patch dependent secretion of maternal IgA into milk. Antigen sampling by M cells in Peyer's-patches are the major source of migratory IgA plasma cells in mammary glands that produce maternal IgA found in milk (59). Similar mechanisms are found in sows with IgA secreted by mammary gland recruited plasma cells exhibiting specificity for antigens in the maternal digestive tract. This “entero-mammary” link is due to the migration of lymphocytes originating in gut associated lymphoid tissue via the bloodstream to the mammary gland (54). Other mechanisms may include viral triggering of goblet cell associated pathways, which present antigens to the immune system and serve as mechanisms of tolerance or translocation outside the gut (60).

In this study 65% of non-GOS piglet fecal samples tested positive for RVA as opposed to 45% for GOS-fed piglet fecal samples representing a significant reduction in infectivity of RVA in the maternally GOS fed group (*P* = 0.008). This reduction in infectivity can be explained by the significantly higher levels of RVA specific IgG and IgA expressed in the GOS fed sows colostrum as possibly modulated by entero-mammary pathways. Nevertheless, there may be other factors affected and/or modulated by GOS feeding such as the many unique proteins, cytokines, exosomes and leucocytes found in sow colostrum (61), which may require further investigation. Previous work has shown that human milk oligosaccharide supplementation can protect pigs against RV infection, as evidenced by shorter diarrhea duration, inhibiting RV binding and/or replication, enhancing mucosal T helper cell and T helper cell 2 cytokine responses and modulating microbiota composition (24). However, this is with direct feeding of GOS to piglets in contrast to the present study where colostrum and then milk were the only source of nutrition for piglets during the study period. In this respect, this study may be one of the first to demonstrate a significant increase in RVA colostrum viral specific immunoglobulins expressed following prebiotic gestational feeding with GOS to sows and concomitant reduction in infectivity in neonates in a commercial farm setting. Out of seventy-one sow fecal samples only seven (9.9%) were RVA positive with no significant difference between non-GOS and GOS fed sows. RVA prevalence rates in pigs varies from 3.3 to 67.3% (2) and prevalence in this study may have been low. Sows are usually immune to RVA, but the virus has been detected in the feces of sows as early as 5 days before farrowing and up to 2 weeks thereafter. Moreover, sows immune to RVA can shed the virus as a result of transient re-infection, or as asymptomatic carriers and at a time when piglets are susceptible to infection (62). Nevertheless, piglets may acquire RVA from their immediate environment given the prevalence of the virus and its stability in feces over time and at ambient temperatures (63). This demonstrates the circulation of RVA from adult sows to piglets and to the environment with resultant re-infection from environmental sources contaminated with RVA positive fecal matter. Animal and environmental RVA reservoirs indicate the need for efficacious detergents that limit the spread and infectivity of RVA and other microbial pathogens as previously described (10, 11) and in this respect GOS supplementation of gestational sows as an adjunct to these practices to reduce the RVA burden in neonates may be useful.

There were no significant differences in α or β-diversity metrics between non-GOS fed sows and GOS fed sows, or piglets born to non-GOS fed sows and piglets born to GOS fed sows. However, highly significant differences in α and β-diversity were seen between non-GOS fed sows and their piglets and GOS fed sows and their piglets (Figures 2, 3) demonstrating major differences in richness, evenness, community membership and structure. Notably, 2.5 times the number of OTUs were recovered from sow fecal samples as opposed to piglet fecal samples. The suckling pig microbiota is particularly different from that of sows and shows a lower bacterial diversity (64). This is not unexpected since piglets have high a high protein and PMO diet compared with the fiber rich diet of sows that support different microbial communities. Moreover, microbial gut diversity increases with age and with longitudinal changes in structure at different growth stages (65). However, it should be considered that both the environment and the sow influence the development of the piglet microbiome. In early lactation, the piglets' GIT microbiota composition is similar to the bacteria found on pen floors, in sow's milk and the nipple surface with the fecal microbiota of piglets becoming more similar to the sow as lactation progresses (66).

Predominant phyla in sows and piglets irrespective of GOS supplementation to sows were *Firmicutes, Bacteroidetes, Proteobacteria*, and *Actinobacteria* in keeping with other studies (65, 67, 68) (Figure 4). However, piglets had a higher relative abundance of taxa at phylum level belonging to *Fusobacteria* (10%) compared with sows (0.3%), which are associated with diarrhea and may be indicative of infection with enteric viruses such as porcine epidemic diarrhea virus which is known to affect the balance of beneficial gut bacteria as opposed to potential bacterial pathogens (69). Irrespective of GOS supplementation, *Clostridium sensu stricto, Acinetobacter, Enterobacteriaceae* unclassified, *Terrisporobacter*, and *Lactobacillus* dominated taxa at genus level in sow fecal samples as did *Bacteroides, Clostridium Senso Stricto, Enterobacteriaceae* unclassified*, Lactobacillus*, and *Streptococcus* in piglet fecal samples (Figure 4). These results were consistent with those from sow and piglet fecal microbiota taken from commercial pig farms (64) and as analyzed by similar methods. However, analyses of differential abundance of taxa at genus level by LEfSe revealed a significant increase in five OTUs belonging to the genus *Treponema* in non-GOS fed sows, but not GOS fed sows (Figure 5). *Treponema* spp are a cause of ear necrosis and shoulder ulcers in pigs leading to animal welfare problems and economic losses for producers (70). LEfSe also indicated a significant and increased differential abundance of *Clostridiales* in both non-GOS sows and non-GOS piglets (Figure 5). Whilst the majority of these organisms are commensal, some have potential to cause severe and sometimes lethal enteric infections in pigs (71). These results may indicate a direct effect of GOS in the sow GIT, thus indicating the capacity for GOS to inhibit pathogen colonization (20, 21). Reduction of *Clostridial* spp in GOS piglets may be explained by piglets inheriting fewer organisms from GOS fed sows with low abundance. Alternatively, sampling and translocation of maternal gut bacteria into colostrum and presentation of antigens to T helper cells by migratory dendritic cells may explain the reduction in *Clostridiales* in piglets (72). In non-GOS fed sows the occurrence of OTUs attributed to *Treponema* and *Clostridia* may indicate that sows harbor potentially pathogenic organisms that may cause pathologies in down-stream production and therefore, GOS supplementation to sows may suppress potential bacterial pathogens in the GIT microbiome, that otherwise may be transmitted allochthonously.

There were no significant differences in microbiota diversity and composition of RVA negative and RVA positive fecal samples taken from piglets when analyzed separately from sow fecal samples (Figure 3). Predominant phyla were *Firmicutes, Bacteroidetes, Proteobacteria, Fusobacteria*, and *Actinobacteria* in keeping with other studies (65, 67, 68) (Figure 4). Abundance of *Fusobacterium* at genus level was higher than that of *Lactobacillus*, which is indicative of viral enteric infection (69). The only OTU occurring at significantly differential levels in RVA negative fecal samples from piglets was *Collinsella* (Figure 5). This bacterium is a member of the *Coriobacteriaceae* and has been strongly and positively correlated with intestinal and circulating rotavirus specific IFN-γ producing CD8+ T helper cell responses, which are known to correlate with protection against rotavirus diarrhea (73). Moreover, *Collinsella* produces ursodeoxycholate which reportedly inhibits binding of SARS-CoV-2 to angiotensin-converting enzyme, suppresses pro-inflammatory cytokines such as TNF-α, IL-1β, IL-2, IL-4, IL-6, and is protective against COVID-19 infection reducing mortality rates (74, 75). *Collinsella* also occurred at significantly differential levels in GOS piglets as opposed to non-GOS piglets although any true link between GOS feeding to gestational sows and occurrence of *Collinsella* in piglets requires further research. In RVA positive piglets two OTUs attributed to *Clostridiales* occurred at significantly differential levels possibly indicating how enteric viruses can favor potential pathogens as opposed to beneficial community members (69, 76) (Figure 5). Indeed, RVA infection favors shifts in ileal microbiome structure with a significant increase in mucin digesting *Bacteroides* as verified by this study in RVA positive piglets (76).

## Conclusions

This study is one of the first to demonstrate that GOS supplementation to sows during gestation significantly increases RVA specific IgG and IgA in colostrum, which confers immunity to neonates and reduces infectivity presumably through the effect of GOS on entero-mammary pathways. The implications for commercial pig farming are that gestational fed GOS could be used as a useful adjunct to other anti-virals and/or cleaning with efficacious detergents that can reduce infectivity in neonates by 20%, which would represent a significant economic gain for commercial herds. Whilst there was no demonstrable effect on microbial diversity of GOS in sows and their offspring, it should be considered that only fecal samples were collected in this study and may not be a true proxy of intestinal contents, which may be different in community membership and structure. In this respect, more research is required. However, non-GOS sows compared with GOS fed sows had a significant and increased differential abundance of potentially pathogenic organisms *Treponema* and *Clostridiales* suggesting GOS modulates the maternal microbiome by suppressing these organisms. The occurrence of *Collinsella* at significantly differential levels in GOS and RVA negative piglet fecal samples as opposed to the occurrence of *Clostridiales* and *Bacteroides* in non-GOS and RVA positive samples suggests modulation of the piglet microbiome through gestational feeding with GOS. Nevertheless, any true link between gestational GOS feeding to sows and occurrence of viral suppressing *Collinsella* in piglets, or indeed any other member of the microbiota requires further research.

## Data availability statement

The datasets presented in this study can be found in online repositories. The names of the repository/repositories and accession number(s) can be found at: https://www.ncbi.nlm.nih.gov/, PRJNA884280.

## Ethics statement

The animal study was reviewed and approved by the Farm Veterinary Consultant and by the University of Nottingham Ethics Committee.

## Author contributions

AL, IC, and KM: conceptualization. AL, LL, and PC: methodology. AL: bioinformatics, formal analyses, and writing–original draft. AL, LL, PC, IC, and KM: validation. AL and KM: investigation. IC and KM: writing–review and editing. IC: funding acquisition. All authors contributed to the article and approved the submitted version.
